# Synthesis of Zinc Oxide Nanoparticles and Their Effect on the Compressive Strength and Setting Time of Self-Compacted Concrete Paste as Cementitious Composites

**DOI:** 10.3390/ijms13044340

**Published:** 2012-04-05

**Authors:** Mohammad Reza Arefi, Saeed Rezaei-Zarchi

**Affiliations:** 1 Department of Civil Engineering and Nano Research Center, Taft Branch, Islamic Azad University, Taft 89915-155, Iran; 2 Department of Biology, Payame Noor University, Yazd 89915-150, Iran; E-Mail: srezaei@ibb.ut.ac.ir

**Keywords:** zinc oxide nanoparticles, cement paste, compressive strength, workability

## Abstract

In the present study, the mechanical properties of self-compacting concrete were investigated after the addition of different amounts of ZnO nanoparticles. The zinc oxide nanoparticles, with an average particle size of about 30 nm, were synthesized and their properties studied with the help of a scanning electron microscope (SEM) and X-ray diffraction. The prepared nanoparticles were partially added to self-compacting concrete at different concentrations (0.05, 0.1, 0.2, 0.5 and 1.0%), and the mechanical (flexural and split tensile) strength of the specimens measured after 7, 14, 21 and 28 days, respectively. The present results have shown that the ZnO nanoparticles were able to improve the flexural strength of self-compacting concrete. The increased ZnO content of more than 0.2% could increase the flexural strength, and the maximum flexural and split tensile strength was observed after the addition of 0.5% nanoparticles. Finally, ZnO nanoparticles could improve the pore structure of the self-compacted concrete and shift the distributed pores to harmless and less-harmful pores, while increasing mechanical strength.

## 1. Introduction

Self-compacting concrete (SCC) is one of the most significant advances in concrete technology in recent years. SCC may be defined as a concrete with the capacity to flow inside the formwork, to pass around reinforcements and through narrow sections, consolidating simply under its own weight without needing additional vibration and without showing segregation or bleeding. To increase the viscosity of the paste, viscosity-modifying admixtures can also be used. These usually comprise polymers made up of long-chain molecules which are capable of absorbing and fixing free water content. This modification in the mix design may have an influence on the mechanical properties of materials; therefore it is important to confirm all of the basic assumptions and test results for design models of SCC construction [1–3].

The use of concrete in constructions and buildings may have begun less than a century ago. However, the increasing the use of concrete from decade to decade has led, much more recently, to extensive and effective research in improvement the properties of concrete, incorporating a wide range of supplementary cementing materials, such as pozzolans and nanoparticles. Recently, nanotechnology has attracted widespread scientific attention because of the new potential uses of particles at the nanometer (10^−9^ m) scale. This may be due to the fact that nanoscale-size particles are able to significantly improve properties compared with grain-size materials of the same chemical composition [4].

There are a small number of studies that have been performed on the incorporation of nanoparticles into SCCs to achieve improved physical and mechanical properties [5]. Nanoparticles can act as heterogeneous nuclei for cement pastes, further accelerating cement hydration, because of their high reactivity, as a nano-reinforcement, and as a nano-filler, making the microstructure denser, and thereby leading to a reduced porosity [6–8]. The most significant issue for all nanoparticles is that of effective dispersion [7–10]. Li *et al*. (2003) investigated the properties of cement mortars, blended with nanoparticles, to explore their super-mechanical and ‘smart’ (temperature and strain sensing) potential [11]. However, until now, research performed over the years has been mainly aimed at achieving high mechanical performance with cement replacement materials at the micro level. There remains a lack of knowledge on the effects of ultra fine and nano-sized particles on the properties of concrete [12].

The aim of the present study was to incorporate the ZnO nanoparticles into SCCs to study the tensile strength, flexural strength and the pore structure of the concrete.

## 2. Results and Discussion

### 2.1. Size Distribution of Synthesized Nanoparticles by SEM and XRD

**Figure 1:**
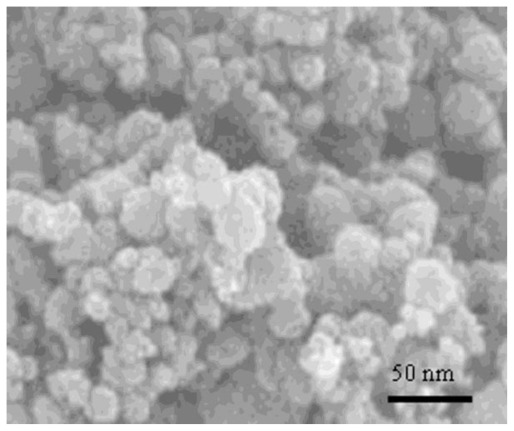
Scanning Electron Microscope image of synthesized ZnO nanoparticles for corresponding sample at 2 h in 160 °C and 2 h in 180 °C.

Figure 1 shows the SEM image of the synthesized ZnO nanoparticles, with an image magnification of about one million. The assembly was attached to a computer running a program to analyze the mean size of the particles in the sample. It should be noted that the particle diameter is always overestimated due to the distortion of SEM images [13].

**Figure 2:**
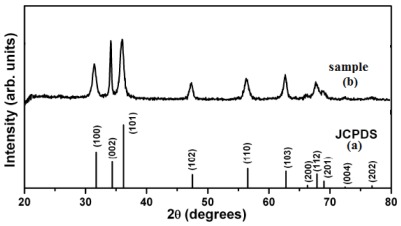
XRD patterns of ZnO nanoparticles: (**a**) standard XRD pattern and (**b**) sample XRD pattern. The results of nanoparticle size measurement of samples by XRD and SEM indicate that the size of the ZnO nanoparticles was about 30 nm.

Figure 2a,b demonstrates the XRD patterns of the synthesized ZnO nanoparticles. The X-ray diffraction data were recorded by using Cu Kα radiation (1.5406 Angstrom). The intensity data were collected over a 2θ range of 20–80°. The average grain size of the samples was estimated with the help of the Scherrer equation, using the diffraction intensity of (101) peak. X-ray diffraction studies confirmed that the synthesized materials were ZnO with wurtzite phase and all the diffraction peaks agreed with the reported JCPDS data; no characteristic peaks were observed other than ZnO. The mean grain size (D) of the particles was determined from the XRD line broadening measurement using the Scherrer Equation (1):

(1) D=0.89λ/(βCosθ)

Where λ is the wavelength (Cu Kα), β is the full width at the half- maximum (FWHM) of the ZnO (101) line and θ is the diffraction angle. A definite line broadening of the diffraction peaks is an indication that the synthesized materials are in the nanometer range. The lattice parameters calculated were also in agreement with the reported values. The reaction temperature greatly influences the particle morphology of as-prepared ZnO powders.

### 2.2. UV-Visible Absorption Spectra of ZnO Nanoparticles

**Figure 3:**
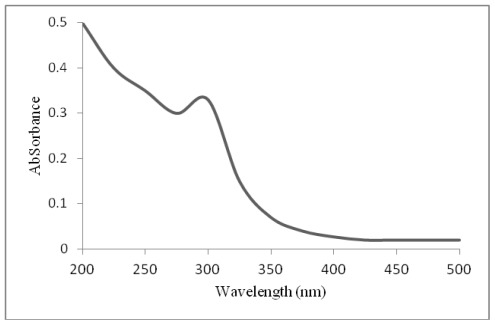
UV-Visible absorption spectra for ZnO nanoparticles.

The UV–visible absorption spectra of ZnO nanoparticles are shown in Figure 3. Although the wavelength of our spectrometer is limited by the light source, the absorption band of the ZnO nanoparticles shows a blue shift due to the quantum confinement of the excitations present in the sample as compared with the bulk ZnO particles. This optical phenomenon indicates that these nanoparticles have a quantum size effect [14].

### 2.3. Analysis of SCC Strength with and without ZnO Nanoparticles

Table 1 shows that by increasing the ZnO content to the concrete paste, the total specific pore volumes of concrete can be decreased and the most probable pore diameters of concretes shift to smaller pores and fall in the range of less-harmful pores or even harmless pores, which indicates that the addition of ZnO nanoparticles improve the structure of concrete.

Table 2 gives the porosities, average diameters and median diameters (volume) of the concrete paste before and after the addition of different proportions of ZnO nanoparticles. The regularity of porosity is similar to that of total specific pore volume. The regularity of average diameter and median diameter (volume) is similar to that of the most probable pore diameter.

During the early stages (week 1) of the hydration process, strength can be affected by the limestone fines that raise the hydration rate of some clinker compounds, since the fines act as nucleation sites of the hydrates formed in the hydration reactions [15]. During the later hydration stages (week 2 or more), it can clearly be seen that there are fewer effects on reducing the flexural strength in SCCs. The pore structure of concrete is the general embodiment of porosity, pore size distribution, pore scale and pore geometry. The addition of ZnO nanoparticles to the concrete paste can help in decreasing its pore size. In terms of the different effects of pore size on concrete performance, a pore size of <20 nm is classified as harmless, while 20–50 nm is thought to be less harmful and 50–200 nm is said to be harmful. A pore size of >200 nm is thought to be highly harmful [16]. In order to analyze and compare conveniently, the pore structure of concrete is divided into four ranges according to this methodology.

### 2.4. Split Tensile Strength

The split tensile strengths of the control and experimental (S1 through S5) mixtures were evaluated with (S1–S5) and without (control) ZnO nanoparticles for 7, 14, 21 and 28 days, as shown in Table 3. Comparison of the results shows that the split tensile strength increased after the addition of ZnO nanoparticles at the concentration of 0.05% of ZnO nanoparticles (S1) after one week (7 days), and the maximum split tensile strength was observed at 0.5% ZnO nanoparticles (S4), as compared to that of the control. The 14, 21 and 28-day experiments did not show significantly different results when compared to those of 7 days.

It was also observed that 1.0% replacement (S5) caused a decrease in the split tensile strength of the experimental cement. This may be due to the fact that the quantity of ZnO nanoparticles present in the mixture was higher than the amount required to combine with the liberated lime during the hydration process, thus leading to excess silica leaching out and causing a deficiency in strength as it replaced a part of the cementing material but did not contribute to its strength [17,18]. It could also be due to defects generated in the dispersion of nanoparticles causing weak zones. The higher split tensile strength in the S4 blended concrete is due to the rapid consumption of Ca(OH)_2_, formed during the hydration of Portland cement, especially at the early stage that can be related to high reactivity of ZnO nanoparticles. As a consequence, the hydration of cement is accelerated and larger volumes of reaction products are formed. It is also thought that ZnO nanoparticles recover the particle packing density of the blended cement, leading to a reduced volume of larger pores in the cement paste [19,20].

### 2.5. Flexural Strength

The flexural strength results of the experimental (S1–S5) and control specimens are shown in Table 4. Similar to the tensile strength, the flexural strength of the specimens increased with the increased percentage of ZnO nanoparticles up to 0.5% replacement (S4) after one week (7 days), and then decreased (S5) when compared to that of the control experiment. It is thought that the increase in flexural strength is due to a rapid consumption of Ca(OH)_2_, which was formed during the hydration process of Portland cement, especially in the early stages, can be related to high reactivity of ZnO nanoparticles. The 14, 21 and 28-day experiments did not show significantly different results when compared to those of 7 days.

The results show that the flexural strength increases by adding 0.5% nanoparticles (S4-SCC series), and then decreases. The addition of 0.5% ZnO nanoparticles produced specimens with much higher flexural strength with respect to all the other experimental specimens and also the control experiment (S0-SCC) of the concrete. The reduced flexural strength produced by adding 1.0% ZnO nanoparticles may be due to the fact that the quantity of ZnO nanoparticles present in the mixture was higher than the amount required to combine with the liberated lime during the process of hydration, thus leading to the leaching out of excess silica and causing a deficiency in strength as it replaces a part of the cementations material but does not contribute to strength. It may also be due to defects generated in the dispersion of nanoparticles, causing weak zones [21,22].

### 2.6. Setting Time

**Figure 4:**
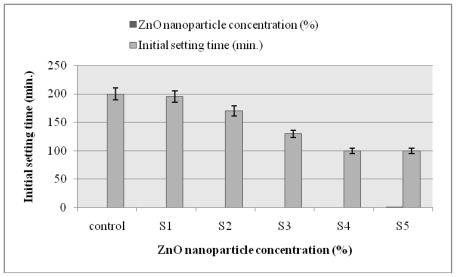
Effect of ZnO nanoparticle concentration on the initial setting time of cement based self-compacted concrete paste.

**Figure 5:**
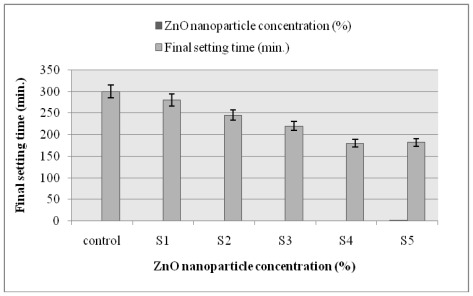
Effect of ZnO nanoparticle concentration on the final setting time of cement-based self-compacted concrete paste.

The experimental results obtained from the initial and final setting times of the cement mortars in the presence of ZnO nanoparticles are shown in Figures 4 and 5, respectively. According to Figures 4 and 5, an increase in the volume fraction of nanoparticles caused a significant decrease in setting time. These results indicate that ZnO nanoparticles have an increased speed of hydration reaction than that of the cement itself. This is because of the phenomenon that ZnO nanoparticles are characterized by their unique surface effects, smaller particle size and higher surface energy [23]. Smaller particle size allows a rapid increase in surface area, leading to a significant and fast rise in the number of superficial atoms. These surface atoms are highly reactive and unstable, which results in a faster reaction speed. Hence, a cautious approach should be taken for the setting time of the cement paste during the utilization of ZnO nanoparticles [23].

The mechanism of the nanoparticles in improving the pore structure of concrete can be attributed to the fact that the nanoparticles are uniformly dispersed in concrete and each particle is contained in a cube pattern. And hence, the distance between nanoparticles can be determined. After hydration begins, hydrated products diffuse and envelop the nanoparticles as kernels. If the content of nanoparticles and the distances between them are appropriate, crystallization will be controlled to a suitable state through restricting the growth of Ca(OH)_2_ crystals by nanoparticles. Moreover, the nanoparticles located in cement paste as kernel can further promote cement hydration due to their high activity. This makes the cement matrix more homogeneous and compact [24,25].

## 3. Experimental Section

### 3.1. Materials

Materials such as zinc oxide ZnO, sodium hydroxide (NaOH), ethanol, potassium nitrate (KNO_3_), potassium hydrogen phosphate (K_2_HPO_4_), potassium hydrogen phosphate (KHPO_4_), acetic acid (CH_3_COOH), sodium acetate (CH_3_COONa), was purchased from Sigma and Merck Co.. All solutions were prepared with double distilled water.

### 3.2. Synthesis of ZnO Nanoparticles

To prepare ZnO nanoparticles, in a typical experiment, a 0.45 M aqueous solution of zinc nitrate (Zn(NO_3_)_2_·4H_2_O) and 0.9 M aqueous solution of sodium hydroxide (NaOH) were prepared in distilled water. Then, the beaker containing NaOH solution was heated to about 55 °C. The Zn(NO_3_)_2_ solutions were added drop wise (slowly for 1 h) to the above heated solution under high-speed stirring. The beaker was sealed at this condition for 2 h. The precipitated ZnO nanoparticles were cleaned with deionized water and ethanol, and then dried in air at about 60 °C. Imaging of the synthesized ZnO nanoparticles was undertaken by a scanning electron microscope, Model XL30- Philips Company, operating at 30 KV.

### 3.3. Experimental Cement

Ordinary Portland Cement (OPC) obtained from the Holcim Cement Manufacturing Company of Malaysia, conforming to ASTM C150 standard was used as received. The chemical and physical properties of the cement are shown in Table 5.

### 3.4. Experimental Self-Compacted Concrete (SCC) Mixture Preparation

A total of two series of mixtures was prepared in the laboratory replicates. The control mixtures were made from the natural aggregates: cement and water. The experimental, or S series (S1–S5), mixtures were prepared with different amounts of ZnO nanoparticles with an average particle size of 30 nm. The experimental mixtures were prepared 0.05, 0.1, 0.2, 0.5 and 1.0% ZnO nanoparticles/cement by weight. The water to binder ratio for all mixtures was set at 0.40 [13]. The aggregates for the mixtures consisted of a combination of crushed basalt and of fine sand, with the sand percentage 30% by weight. The binder content of all mixtures was 550 kg/m^3^.

### 3.5. Preparation of Test Specimens

Series S mixtures were prepared by mixing the course aggregates, fine aggregates and powdered materials (cement and ZnO nanoparticles) in a laboratory concrete drum mixer. The powdered material was only cement. They were mixed in dry condition for two minutes, and for another three minutes after adding the water. Samples of the fresh concrete were determined immediately to evaluate flexural strength following the mixing procedure. Cylinders with a diameter of 150 mm and a height of 300 mm for split tensile strength and cubes with 200 mm × 50 mm × 50 mm edges for flexural strength tests were cast and compacted in two layers on a vibrating table, with each layer being vibrated for 10 s [14]. The molds were covered with polyethylene sheets and moistened for 24 h. Then, the specimens were demolded and cured in water at 20 °C prior to the experimental period. The tensile strength tests of the concrete samples were determined at 7 and 14 days. The reported results were the average of three replicates.

### 3.6. Split Tensile Strength of ZnO Nanoparticles Blended Self-Compacted Concrete

A split tensile test was carried out in accordance to the ASTM C 496-90 standard. After the specified curing period, the concrete cylinders were subjected to split tensile test by using a universal testing machine. Tests were carried out on triplicate specimens and average split tensile strength values were obtained.

### 3.7. Flexural Strength of ZnO Nanoparticle-Modified Self-Compacted Concrete

The flexural strength of concrete is used as a structural design criterion and as a general indicator of concrete strength. ASTM C 293 (C-293) determines the flexural strength of concrete specimens by the use of a simple beam with center-point loading. Flexural tests were undertaken in accordance with the ASTM C293 Standard. Similar to the tensile tests, flexural tests were carried out on triplicate specimens and average flexural strength values were obtained.

### 3.8. Setting Time of ZnO Nanoparticle-Modified Self-Compacted Concrete

Setting time of the specimens was regulated according to the ASTM C191 standard. The ASTM C191 method determines the time of setting of hydraulic cement by the means of the Vicat needle.

## 4. Conclusions

The ZnO nanoparticle blended concrete had higher split tensile and flexural strengths as compared to that of the control experiment. It is found that cement could be advantageously replaced with ZnO nanoparticles and the optimum level of ZnO nanoparticle content was achieved with a 0.5% replacement. On the whole, the addition of nanoparticles improves the pore structure and mechanical strength of concrete. On the one hand, nanoparticles can act as a filler to enhance the density of concrete, which leads to the porosity of concrete being significantly reduced. On the other hand, nanoparticles can not only act as an activator to accelerate cement hydration due to their high activity, but also act as a kernel in cement paste, which makes the size of Ca(OH)_2_ crystal smaller and increases the chance of tropism to occur.

## Figures and Tables

**Table 1:** Total specific pore volumes and most probable pore diameters of S0-SCC and S(1–5)SCC specimens, before and after the addition of different proportions of ZnO nanoparticles.

Sample Designation	Total Specific Pore Volume (mL/g)	Most Probable Pore Diameter (nm)
S0-SCC (Control)	0.040	67
S1-SCC (0.05% ZnO)	0.038	51
S2-SCC (0.1% ZnO)	0.0356	43
S3-SCC (0.2% ZnO)	0.0321	32
S4-SCC (0.5% ZnO)	0.0301	21
S5-SCC (0.1% ZnO)	0.0296	14

**Table 2:** Porosities, average diameters and median diameters (volume) of S0-SCC and S(1–5)SCC specimens, before and after the addition of different proportions of ZnO nanoparticles.

Sample Designation	Porosity (%)	Average Diameter (nm)	Median Diameter, Volume (nm)
S0-SCC (Control)	10.2	31.56	45.00
S1-SCC (0.05% ZnO)	9.66	29.63	37.5
S2-SCC (0.1% ZnO)	9.02	26.81	31.5
S3-SCC (0.2% ZnO)	8.51	21.30	26.0
S4-SCC (0.5% ZnO)	7.93	17.63	21.0
S5-SCC (0.1% ZnO)	6.34	14.97	19.50

**Table 3:** Split tensile strength of self-compacted concrete, before and after the addition of different concentrations of ZnO nanoparticles.

Sample Designation	ZnO Nanoparticles (%)	Split Tensile Strength (MPa)			

		7 Days	14 Day	21 Days	28 Days
Control	0	4.7	5.0	5.1	5.0
S1	0.05	5.2	5.4	5.3	5.1
S2	0.1	5.8	5.7	5.3	5.0
S3	0.2	6.5	6.55	5.4	5.2
S4	0.5	7.0	6.6	5.2	5.3
S5	1.0	6.6	6.5	5.3	5.2

Water was used to make the cement paste + ZnO nanoparticles with the ratio of 0.40.

**Table 4:** Flexural strength of self-compacted concrete, before and after the addition of different concentrations of ZnO nanoparticles.

Sample Designation	ZnO Nanoparticles (%)	Split Tensile Strength (MPa)			

		7 Days	14 Day	21 Days	28 Days
Control	0	2.0	2.3	2.1	2.0
S1	0.05	2.3	2.4	2.3	2.1
S2	0.1	2.8	2.7	2.3	2.0
S3	0.2	3.1	3.2	2.4	2.2
S4	0.5	4.0	3.6	2.2	2.3
S5	1.0	3.0	3.5	2.3	2.2

Water was used to make the cement paste + ZnO nanoparticles with the ratio of 0.40.

**Table 5:** Chemical and physical properties of Portland cement (Wt%).

	Chemical Properties				
**Material**	SiO_2_	Al_2_O_3_	Fe_2_O_3_	CaO MgO	
**Cement**	21.89	5.3	3.34	53.27	6.45
**Material**	SO_3_	Na_2_O	K_2_O	Loss On Ignition	
**Cement**	3.67	0.18	0.98	3.21	

Specific Gravity: 1.7 g/cm^3^.
